# Lipopolysaccharide, Identified Using an Antibody and by PAS Staining, Is Associated With *Corpora amylacea* and White Matter Injury in Alzheimer's Disease and Aging Brain

**DOI:** 10.3389/fnagi.2021.705594

**Published:** 2021-11-24

**Authors:** Xinhua Zhan, Marisa Hakoupian, Lee-Way Jin, Frank R. Sharp

**Affiliations:** ^1^Department of Neurology, University of California Davis School of Medicine, Sacramento, CA, United States; ^2^Department of Pathology, University of California Davis School of Medicine, Sacramento, CA, United States

**Keywords:** lipopolysaccharide vesicles, *Corpora amylacea*, white matter injury, Alzheimer's disease, neuroinflammation, innate immune response, astrocytes, human brain

## Abstract

*Corpora amylacea* (CA) increase in number and size with aging. Their origins and functions remain unknown. Previously, we found that Alzheimer's disease (AD) brains have more *CA* in the periventricular white matter (PVWM) compared to aging controls. In addition, CA is associated with neurodegeneration as indicated by colocalization of degraded myelin basic protein (dMBP) with periodic acid-Schiff (PAS), a CA marker. We also found that bacterial lipopolysaccharide is present in aging brains, with more LPS in AD compared with controls. Periodic acid-Schiff staining is used to identify CA by virtue of their high polysaccharide content. Despite the growing knowledge of CA as a contributor to AD pathology, the molecules that contribute to the polysaccharides in CA are not known. Notably, lipopolysaccharides (LPS) are important cell-surface polysaccharides found in all Gram-negative bacteria. However, it is unknown whether PAS could detect LPS, whether the LPS found in aging brains contribute to the polysaccharide found in CA, and whether LPS associate with myelin injury. In this study, we found that aging brains had a myelin deficit zone (MDZ) adjacent to the ventricles in PVWM. The MDZ contained vesicles, most of which were CA. LPS and dMBP levels were higher in AD than in control brains. LPS was colocalized with dMBP in the vesicles/CA, linking white matter injury with a bacterial pro-inflammatory molecule. The vesicles also contained oxidized fibers, C-reactive protein, NG2, and GALC, markers of oligodendrocyte precursor cells (OPCs) and oligodendrocyte cells (OLs), respectively. The vesicles/CA were surrounded by dense astrocyte processes in control and AD brains. LPS was co-localized with CA by double staining of PAS with LPS in aging brains. The relationship of LPS with PAS staining was confirmed by PAS staining of purified LPS on nitrocellulose membranes. These findings reveal that LPS is one of the polysaccharides found in CA which can be stained with PAS. In addition, vesicles/CA are associated with oxidized and damaged myelin. The LPS in these vesicles/CA may have contributed to this oxidative myelin damage and may have contributed to oxidative stress to OPCs and OLs which could impair the ability to repair damaged myelin in AD and control brains.

## Introduction

*Corpora amylacea* are glycoprotein-containing inclusions found in the aging brain and other organs. *Corpora amylacea* (CA) were not considered to be of any pathological significance for more than a century. However, recently, CA are reported to be associated with various neurodegenerative diseases including Alzheimer's disease (AD) (Averback, 1981; Tate-Ostroff et al., 1989; Cisse et al., 1993; Singhrao et al., 1993; Renkawek and Bosman, 1995; Zhan et al., 2014), multiple sclerosis (Gati and Leel-Ossy, 2001; Selmaj et al., 2008), amyotrophic lateral sclerosis (Atsumi, 1981; Gati and Leel-Ossy, 2001), Parkinson's disease (Buervenich et al., 2001; Pisa et al., 2016), and Huntington's disease (Averback, 1981). CA usually increase in number with advanced age in normal human brains.

*Corpora amylacea* are positive for periodic acid-Schiff (PAS) staining due to their high polysaccharide content. Besides containing glucose polymers, many other components derived from the breakdown products of neurons, oligodendrocytes, and astrocytes are reported (Ramsey, 1965; Anzil et al., 1974; Palmucci et al., 1982; Singhrao et al., 1993, 1994; Leel-Ossy, 2001; Auge et al., 2019). In addition, components from blood plasma, blood cells, and other cells of the human body are identified in the CA of human brains. These components include ubiquitin (Sahlas et al., 2002), heme oxygenase-1 (Sahlas et al., 2002), thrombospondin (Meng et al., 2009), complement (Singhrao et al., 1995), S100 proteins (Hoyaux et al., 2000), and calprotectin, a soluble protein contained in neutrophil granules. Calprotectin is a mammalian antimicrobial protein that has antibacterial and antifungal properties. Recently, pathological structures associated with fungal infections have been demonstrated in CA in human brains (Pisa et al., 2016, 2018). In addition, a recent proteomic analysis identified several peptides associated with Gram-negative and lipopolysaccharide (Peters et al., 2005) containing proteobacteria in CA (Pisa et al., 2018). These findings suggest that CA are not just inert structures that occur in the brain but may be part of an orchestrated immune system response that involves vascular components, damage to white matter and adjacent brain tissues, and microbial components including Gram negative bacteria.

We have previously reported that neuronal and myelin breakdown products, including dMBP, neurofilament, and myelin lipids are detected in CA of aging brains. We have also reported that AD brains have more CA compared to control aging brains (Zhan et al., 2014). AD brains have more dMBP^+^ vesicles in the periventricular white matter (PVWM) compared to controls (Zhan et al., 2014). The ependymal cells that separate the ventricles from PVWM are denuded in aging brains, with the ependymal damage being more extensive in the AD brain compared to controls (Zhan et al., 2014). We also found that bacterial components including lipopolysaccharides (LPS) are present in AD and aging brains with much more LPS in AD compared to controls (Zhan et al., 2016). LPS was localized to neurons, oligodendrocytes, oligodendrocyte progenitor cells (OPCs), microglia, and ependymal cells (Zhan et al., 2016). LPS also localized to virtually all amyloid plaques in AD brains. These findings show that several features of AD pathology associate with the presence of bacterial LPS, and raise the question of whether LPS contribute to AD pathology, or whether AD pathology leads to the accumulation of LPS.

Lipopolysaccharides are pro-inflammatory molecules that can elicit a potent innate immune response and lead to the production of cytokines and inflammation that could contribute to the formation of PAS positive vesicles/CA that associate with damaged myelin. Thus, we determined that PAS positive vesicles/CA that contained dMBP also contained LPS, and that dMBP and LPS levels were higher in AD compared to control brains. We also showed significantly more LPS^+^ CA/vesicles in AD compared to control brains. Since an antibody was used to detect LPS immunocytochemically, we used an independent enzymatic Limulus Amoebocyte Lysate (LAL) assay to also show higher LPS activity levels in AD compared with control brains. Since PAS stains polysaccharides and LPS are cell-surface polysaccharides found in all Gram-negative bacteria, we performed double staining of LPS and PAS in AD and aging brains. The association of LPS and PAS was further confirmed by PAS staining of purified LPS on nitrocellulose membranes. The relationship between LPS^+^ and PAS positive vesicles/CA to oligodendrocytes, OPCs, oxidative stress markers, C-reactive protein (CRP), microglia, and astrocytes are also shown.

## Materials and Methods

### Brain Samples

Postmortem brain samples were provided by the Alzheimer's Disease Center at the University of California Davis (UCD ADC). The study was approved by the UCD Institutional Review Board. A written informed consent to share research tissues after death was obtained from all participants or their proxy prior to their death. The clinical diagnosis of AD was made by the board-certified neurologists and pathological diagnosis was confirmed by the board-certified neuropathologists. AD pathology was rated using Consortium to Establish a Registry for Alzheimer's Disease (CERAD) criteria and staging of Braak. Controls were normal individuals, those with a low likelihood of clinical AD and individuals who did not meet the criteria for AD neuropathology. Controls were matched to AD based on age and sex. A total of 50 brains, including 30 AD and 20 control brains were studied. Blocks of tissue including frontal PVWM at the level of the head of the caudate nucleus from each brain were removed. For immunostaining, brains were fixed in formalin and brain tissue was embedded in paraffin. Sections were cut in the coronal plane. For Western blot analyses and the LAL assays, the brain tissue was frozen at −70°C.

### Immunohistochemistry

Detailed methods are described in our previous studies (Zhan et al., 2008, 2014, 2015). Briefly, after removing paraffin with xylene (2 min × 3 times) and rehydrating through graded alcohols (2 min in 100% × 2 times; 2 min in 95% and 2 min in 75%), brain sections were incubated in endotoxin-free 0.1M PBS antigen retrieval buffer containing 1 mM EDTA and 0.05% Tween 20 at 95°C for 20 min. Endogenous peroxidase activity was quenched with 3% H_2_O_2_ in endotoxin-free PBS for 20 min. The endotoxin-free 0.1M PBS blocking buffer contained 2% goat serum, 1% BSA, and 0.3% Triton 100.

Primary antibodies used in immunohistochemistry included mouse monoclonal antibodies against Gram-negative bacterial LPS (MD-05-0148, RayBiotech, Norcross, GA, USA), MBP (MAB382, Millipore, Burlington, MA, USA), rabbit polyclonal antibodies against 8-Iso-PGF2α (ADI-905-015-100, Enzo Biochem, Farmingdale, NY, USA), and 13-14-DH-15keto- PGF2α (ADI-905-051-100, Enzo Biochem). The secondary antibody was a biotinylated goat anti-mouse IgG (1:200 dilution, Vector Labs, Burlingame, CA, USA). The antibody complex was detected using ABC reagent, alkaline phosphatase (AP), Vector^®^ Blue Substrate Kit, or a Horseradish peroxidase (HRP), and Vector^®^ VIP Substrate Kit or DAB Substrate Kit according to the instructions of the manufacturer (Vector Labs). The primary antibody was omitted to assess non-specific staining.

### Immunofluorescence

Immunofluorescence methods are described in our previous studies (Zhan et al., 2008, 2014, 2015). Briefly, after removing paraffin, rehydrating, and antigen retrieval, as mentioned above, sections were treated with Autofluorescence Eliminator Reagent (2160, Millipore). The primary antibodies included mouse monoclonals against myelin basic protein (MBP) (MBP382, Millipore), neuron-glial antigen 2 (NG2) (MAB5384, Millipore), galactocerebroside (GALC) (MAB342, Millipore), Gram-negative bacterial LPS (MD-05-0148, Ray Biotech), and rabbit polyclonal antibodies against dMBP (AB5864, Millipore), 8-Iso-PGF2α (ADI-905-015-100, Enzo Biochem), 13-14-DH-15keto- PGF2α (ADI-905-051-100, Enzo Biochem), CRP (PA1-29087, Thermo Fisher, Waltham, MA, USA), Iba1 (019-19741, Wako, Richmond, VA, USA), and glial fibrillary acidic protein (GFAP) (250661, Invitrogen, Waltham, MA, USA). Goat anti-mouse or goat anti-rabbit Alexa Fluor^®^ 488 or 594 conjugated antibodies (Invitrogen) were used for secondary antibodies depending on the species of the primary antibody. Slides were cover slipped with a mounting medium containing DAPI and examined under a Nikon Eclipse E600 fluorescent microscope at excitation/emission wavelengths of 493/520 nm (for green fluorochrome), 590/619 nm (for red fluorochrome), or 358/463 nm (for blue fluorochrome). For controls, the primary antibody was deleted or immunodepleted with the target antigen of the antibody.

Autofluorescence occurs in aging brains, which can interfere with the detection of specific fluorescent signals and can be problematic if not removed. We treated all sections with Autofluorescence Eliminator Reagent prior to immunostaining, which eliminated most autofluorescence in both control and AD brains (Supplementary Figure 1). These results suggest that the immunofluorescent signals detected in this study were not due to autofluorescence.

The specificity of mouse monoclonal antibody against bacterial LPS was verified, previously (Zhan et al., 2016, 2018). Immunostaining controls for the other antibodies were carried out by omitting the primary antibodies in control and AD brain sections. The results showed that none of the vesicles and other brain structures were stained positively (Supplementary Figure 2). These results suggest that the staining was not due to the non-specific binding of the secondary antibodies used.

### Western Blot Analysis

Detailed methods are described in our previous studies (Zhan et al., 2008, 2014, 2015). Briefly, frozen tissues were homogenized in ice-cold RIPA buffer containing a complete protease inhibitor (Sigma). Homogenates were centrifuged at 14,000 × *g* for 30 min at 4°C. Protein (12.5 μg each) from the supernatant was loaded on 7.5% sodium dodecyl sulfate (SDS) polyacrylamide gels and transferred to the nitrocellulose membrane. The primary antibody included rabbit polyclonal against dMBP (AB5864, 1:1,000 dilutions; Millipore). NIH Image J software was used to quantify band intensities. A mouse monoclonal against β-actin (sc-69879, Santa Cruz, Dallas, TX, USA) was used as a loading control for Western blots and optical densities of each target protein normalized to β-actin. Horseradish peroxidase (HRP) conjugated anti-mouse or anti-rabbit IgG (Bio-rad) was used to detect the primary antibody. The ECL chemiluminescent detection system (PIERCE Inc., Thermofisher Scientific, Waltham, MA, USA) was used to detect the signals. Blots were imaged on the Fluorchem 8900 system (Alpha Innotech, San Leandro, CA, USA). The ratio of the intensity of dMBP/β-actin bands was quantified with NIH Image J software. The relative band intensity in AD samples was averaged and compared to the averaged band intensity of control samples.

### Limulus Amoebocyte Lysate Assay

The LAL enzymatic assay for LPS was performed using an Endpoint Chromogenic LAL Assay kit (50-647U, Lonza) according to the instruction of the manufacturer. A standard curve was generated from known amounts of LPS, and this curve was used to derive the values from brain samples. The LAL enzymatic assay for LPS was performed to confirm the results obtained using the monoclonal antibody to LPS.

### Quantitative Analysis of LPS^+^ Vesicles

Sections including frontal PVWM at the level of the head of the caudate nucleus from AD and control brains were used for counting LPS^+^ vesicles. Three sections per brain were counted. The average of number of three brain sections in each case was used for the statistical analysis. Only clearly stained round vesicles were counted. PVWM was defined as the white matter within 1 mm of the ependymal layer. The numbers of vesicles were counted in random areas about 0.8 mm^2^ (20X fields) by an investigator blinded to diagnosis using NIH Image J software.

### PAS Staining of CA and Purified LPS

Brain sections with PVWM were incubated in 0.5% periodic acid (Sigma) for 5 min at room temperature followed by washing in tap water for 1 min. Sections were then incubated in Schiff reagent (Sigma) for 10 min followed by washing in tap water for 10 min. Sections were mounted with an aqueous mounting medium and cover slipped.

To further confirm that PAS stains LPS, purified LPS from *E. coli*, serotype O111:B4 (L2630, Sigma-Aldrich, St. Louis, MO, USA) and its mutant form of *E. coli*, serotype J5 (ALX-581-014-L002, Enzo Biochem) were used for PAS staining. LPS from *E. coli* O111:B4 was purified by phenol extraction and dissolved in endotoxin free water. LPS from *E. coli* J5 is purified by a modification of the PCP extraction and dissolved in sterile pyrogen-free double distilled water. Bovine serum albumin (BSA, 23209, Pierce™, Thermofisher Scientific, Waltham, MA, USA) was used as a control. Three microliters of samples from each concentration of LPS and BSA were spotted onto the nitrocellulose membrane and air dried for 30 min. The membrane was washed with PBS followed by 0.5% periodic acid (Sigma) for 5 min at room temperature before washing in PBS. The membrane was then incubated in Schiff reagent (Sigma-Aldrich) for 10 min followed by washing in PBS. The intensity of PAS staining was measured using NIH Image J software.

### Statistical Analyses

Differences between AD and control groups were analyzed using Student's *t*-test (continuous), Kruskal-Wallis test (ordinal), and Fisher Exact test (categorical). Differences among *E. coli* serotype J5 LPS, *E. coli* O111:B4 LPS, and BSA groups were analyzed using One Way ANOVA (SigmaStat). Values were expressed as mean ± SE. A *p* ≤ 0.05 was considered significant.

## Results

### Patient Characteristics

Characteristics of the 30 patients with AD and 20 control patients are shown in Table 1. There were no differences for controls compared to AD in age or sex. The differences in median Braak and Braak stage and CERAD between AD and control brains were significant, as expected.

**Table 1:** Demographic data and neuropathological assessment of Alzheimer's disease and control patients.

	**Controls (*n* = 20)**	**AD (*n* = 30)**	***p*-values**
Age (years ± SE)	83.6 ± 1.4	80.3 ± 1.6	0.078
Sex male: n (%)	9 (45.0)	12 (40.0)	0.73
Braak stage: median	2 (IQR 1, 2)	6 (IQR 5, 6)	<0.001
CERAD: median	0 (IQR 0, 1)	3 (IQR 3, 3)	<0.001

*Differences between groups were analyzed suing a Student t-test (continuous), Kruskal-Wallis test (ordinal) or Fisher Exact test (categorical). IQR, interquartile range*.

### Periventricular Myelin Is Deficient in Aging Control and AD Brain

Our previous studies showed the presence of dMBP in both control and AD brains, though there was more dMBP in AD brains (Zhan et al., 2014). Since it was unclear where the dMBP was coming from, we stained the control and AD brains with an antibody specific for intact MBP.

**Figure 1:**
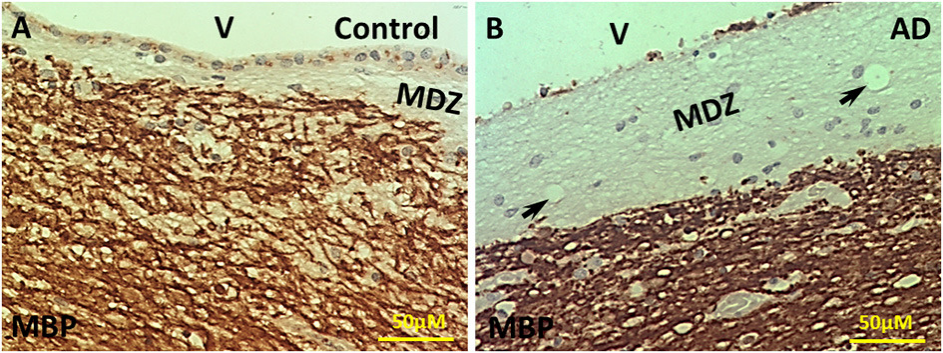
Immunohistochemistry of MBP in the PVWM of AD and control brains. **(A)** Intact MBP immunostaining in the PVWM of control brain. A thin myelin-deficit zone (MDZ) was observed under the ependymal cells. Some ependymal cells were stained for Intact MBP. **(B)** Intact MBP staining in the PVWM of AD brain. The MDZ was much wider in AD brain. There was a loss of many ependymal cells. A few areas of no tissue staining were observed (arrows). Note: Dark Brown, positive staining; MBP, myelin basic protein; V, ventricle; MDZ, myelin-deficient zone; PVWM, periventricular white matter; AD, Alzheimer's disease; Bar = 50 μm.

In control aging brains, there was intense MBP immunostaining in the PVWM except for a thin myelin-deficit zone (MDZ, Figure 1A). The width of the myelin-deficit zone in controls varied between 25-150 μm with an intact ependymal lining separating the ventricle (V) from the MDZ. Some ependymal cells stained positive for MBP (Figure 1A). In AD brains, the myelin-deficient zone was wider and varied between 75 and 450 μm (Figure 1B). Occasional clear vesicles were observed in the myelin-deficit zone in the AD brain (Figure 1B, arrows). Thus, we deduced that one source of dMBP in both control and AD brains was in the zone between the ependyma and intact myelin adjacent to the ventricle in the area we termed the “myelin-deficient zone” (MDZ).

### Oxidized Vesicle Walls

Since there is evidence for increased oxidative stress in normal aging and AD brain, and since oxidative stress might play a role in the loss of myelin in the MDZ, we stained the control and AD brains for two different but related oxidative stress markers.

**Figure 2:**
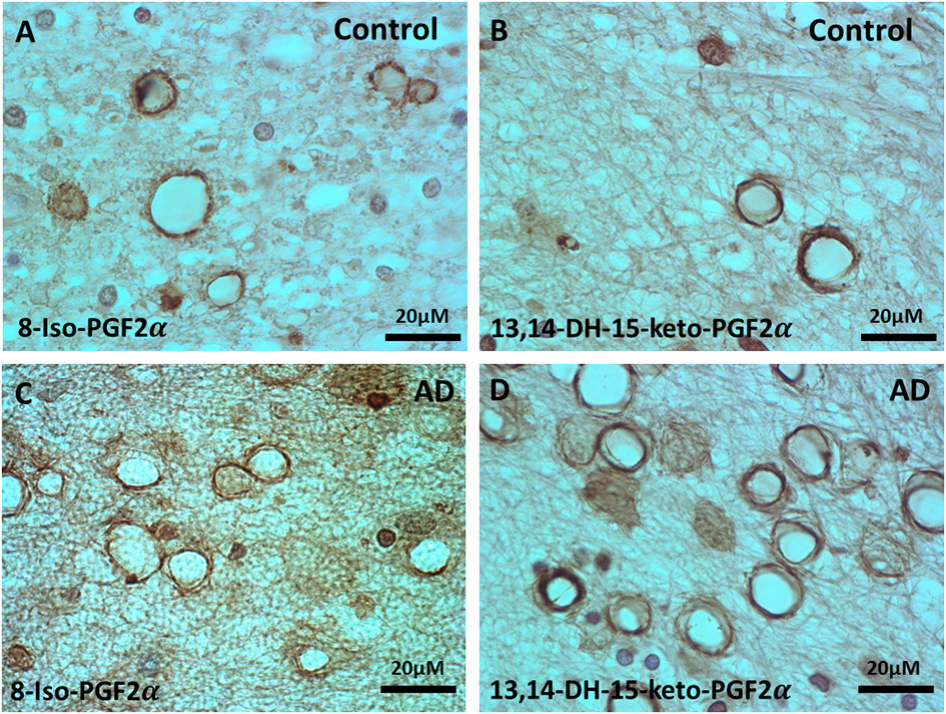
Immunohistochemistry of oxidative markers, 8-Iso-PGF2α and PGFM, in the vesicles of control and AD brains. **(A)** 8-Iso-PGF2α stained vesicles of PVWM in control brain. **(B)** PGFM, also known as 13,14,-Dihydro-15-keto-PGF2α, stained vesicles in the PVWM of the control brain. **(C)** 8-Iso-PGF2α stained vesicles in AD PVWM. **(D)** PGFM stained vesicles in AD PVWM. Note: PGFM, Prostaglandin F2α metabolite; 8-Iso-PGF2α, 8-Iso-Prostaglandin F2α; PVWM, periventricular white matter; AD, Alzheimer's disease; Bar = 20 μm.

Staining for oxidative stress markers was performed in the MDZ in PVWM of control (Figures 2A,B) and AD brains (Figures 2C,D). The staining revealed vesicles that stained for 8-Iso-PDG2α in the control (Figure 2A) and AD brains (Figure 2C), and stained for PGFM, also known as 13,14,-Dihydro-15-keto-PGF2α, in control (Figure 2B) and AD brains (Figure 2D). Staining for the majority of vesicles was in the walls with a hollow center, though some of the vesicles were full of oxidized filaments (Figure 2). Vesicles varied in size from ~4 to ~20 μm in diameter (Figure 2). Thus, there is evidence of oxidative stress in the MDZ as manifested by vesicles that we have previously noted to be positive for dMBP.

### Oxidized NG2 and GALC in the Vesicles

Since the oxidatively stressed vesicles occurred in the region of myelin loss adjacent to the ventricles, we reasoned that the vesicles might be made up partially from damaged oligodendrocytes and oligodendrocyte progenitor cells (OPCs). Thus, we stained sections for GALC and NG2, oligodendrocyte, and OPC markers, respectively.

**Figure 3:**
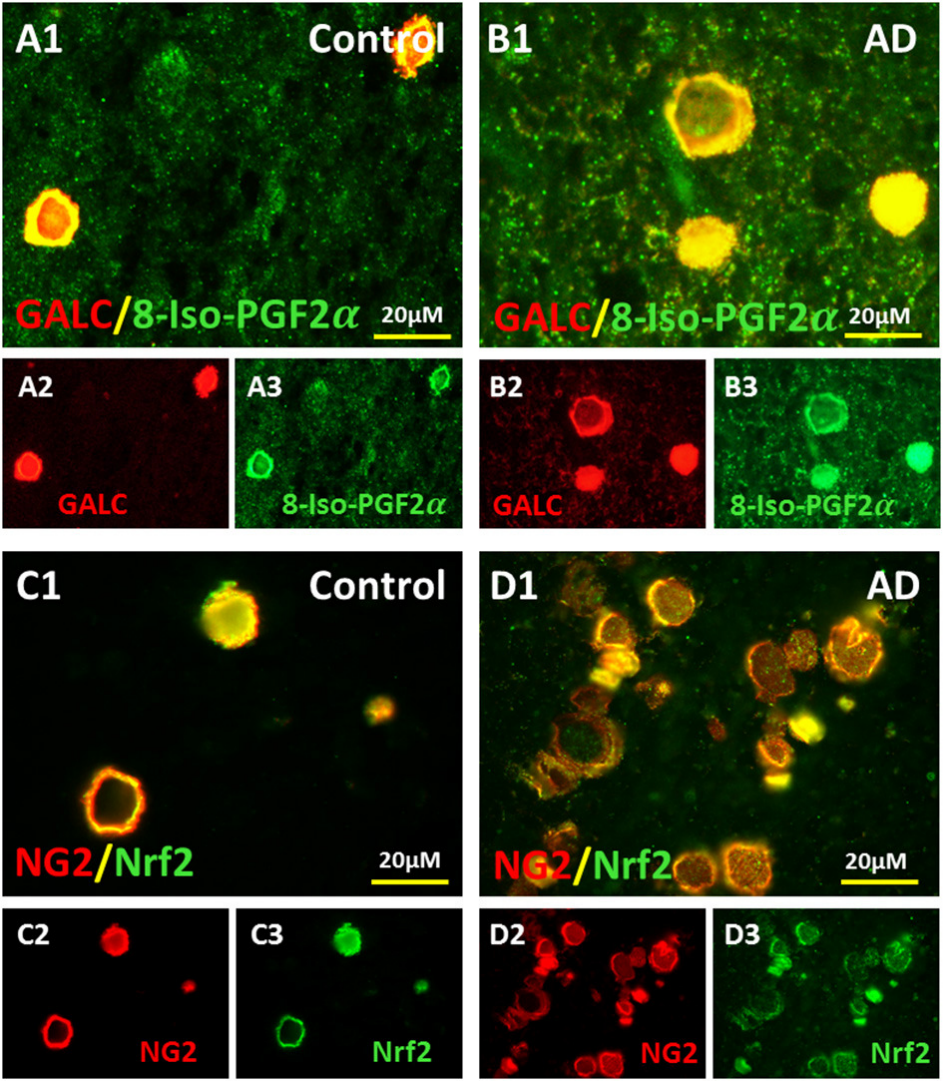
Colocalization of OLs and OPCs markers with oxidative markers in the vesicles of AD and aging brains. Oligodendrocyte marker, GALC, in control **(A2)** and AD brains **(B2)** and 8-Iso-PGF2α in control **(A3)** and AD brains **(B3)** colocalized in control **(A1)** and AD brains **(B1)**. Oligodendrocyte precursor cell (OPCs) marker, NG2, in control **(C2)** and AD brains **(D2)** and oxidative stress marker Nrf2 in control **(C3)** and AD brains **(D)** colocalized in control **(C1)** and AD brains **(D1)**. Note: OL, oligodendrocyte; OPC, oligodendrocyte precursor cell; GALC, Galactocerebroside; NG2, neuron-glial antigen 2; Nrf2, Nuclear factor erythroid 2-related factor 2; AD, Alzheimer's disease; Bar = 20 μm.

The oligodendrocyte marker, Galactocerebroside (GALC), was localized to vesicles in the MDZ of control brains (Figures 3A2,A1) and AD brains (Figures 3B2,B1), and co-localized with the oxidative stress marker 8-Iso-PGF2α in control (Figures 3A1,A3) and AD brain (Figures 3B1,B3). Similarly, the oligodendrocyte precursor cells (OPCs) marker, neuron-glial antigen 2 (NG2), was localized to vesicles in the MDZ of control brains (Figures 3C2,C1) and AD brains (Figures 3D2,D1), and co-localized with oxidative stress marker Nrf2 in control brain (Figures 3C1,C3) and AD brain (Figures 3D1,D3). Thus, oligodendrocyte and OPC markers are in the vesicle walls in the MDZ, and both markers are associated with oxidative stress.

### Detection of Bacterial LPS in the Vesicles

We have previously shown that LPS, the polysaccharide found in the outer wall of all Gram-negative bacteria, is found in both control and AD brain, though there are much more LPS in AD brain. We wondered if one cause for the increased oxidative stress in the MDZ might be related to LPS.

**Figure 4:**
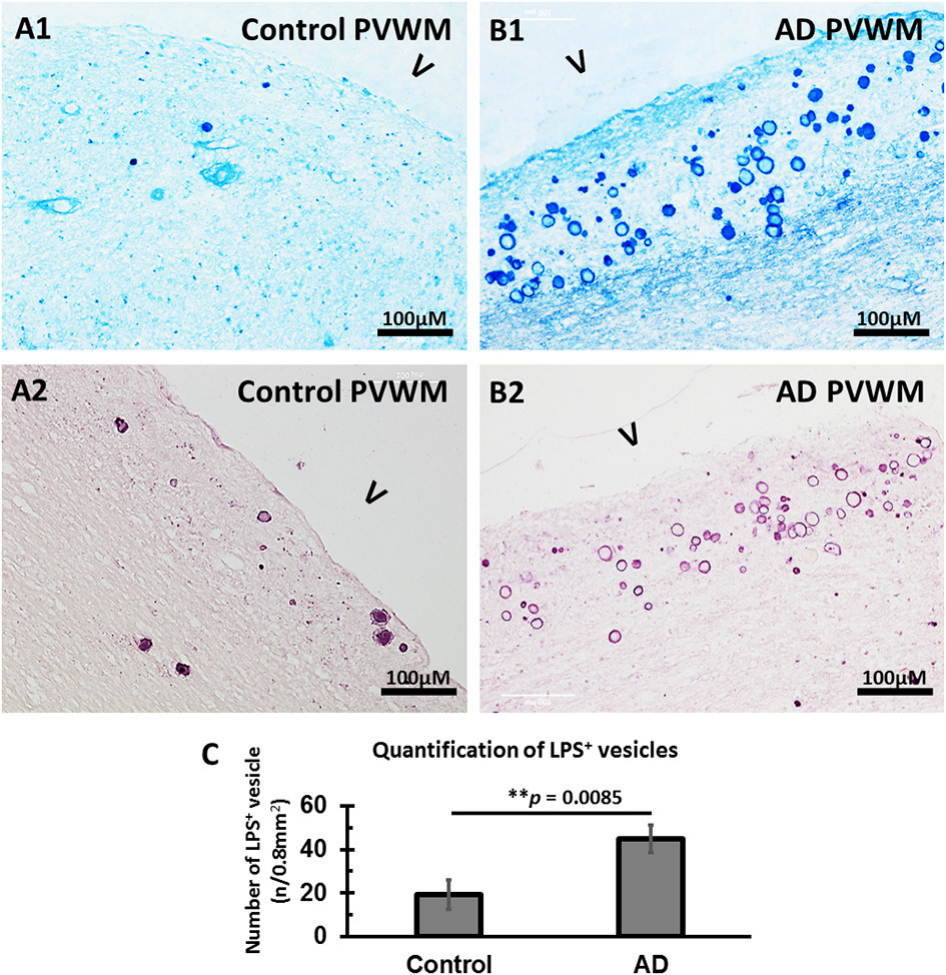
Immunostained LPS vesicles in the PVWM of AD and aging control brains. **(A)** LPS was identified in the vesicles in control PVWM using an antibody against LPS and staining with alkaline phosphatase and Vector® Blue Substrate Kit **(A1)** or horseradish peroxidase and Vector VIP Substrate Kit **(A2)**. **(B)** LPS was identified in the vesicles in AD PVWM using an antibody against LPS and staining with alkaline phosphatase and Vector Blue Substrate Kit **(B1)** or horseradish peroxidase and Vector VIP Substrate Kit **(B2)**. **(C)** Quantitative analysis showed more LPS^+^ vesicles in AD (44.8 ± 6.5, *n* = 12) PVWM compared to control PVWM (20.1 ± 6.7, *n* = 9, *p* = 0.0085). Note: LPS, lipopolysaccharide; V, ventricle; PVWM, periventricular white matter; AD, Alzheimer's disease; Bar = 100 μm.

Staining for LPS in the myelin-deficient zone (MDZ) of the control brain (Figures 4A1,A2) showed some LPS stained vesicles. In contrast, there were many more LPS stained vesicles in the MDZ of the AD brain (Figures 4B1,B2). A quantification confirmed this with more LPS^+^ stained vesicles in the myelin-deficit zone of AD brains (44.8 ± 6.5) compared to controls (20.1 ± 6.7, *p* = 0.0085) (Figure 4C). Thus, LPS were also found in the walls of vesicles in the MDZ of control and AD brain and could contribute to the oxidative stress associated with the vesicles.

### Association of LPS With Myelin Degradation Within the Vesicles

We have previously shown that dMBP was associated with vesicles in the MDZ of the PVWM (Zhan et al., 2014). We reasoned that dMBP might co-localize with LPS in the vesicles in the MDZ.

**Figure 5:**
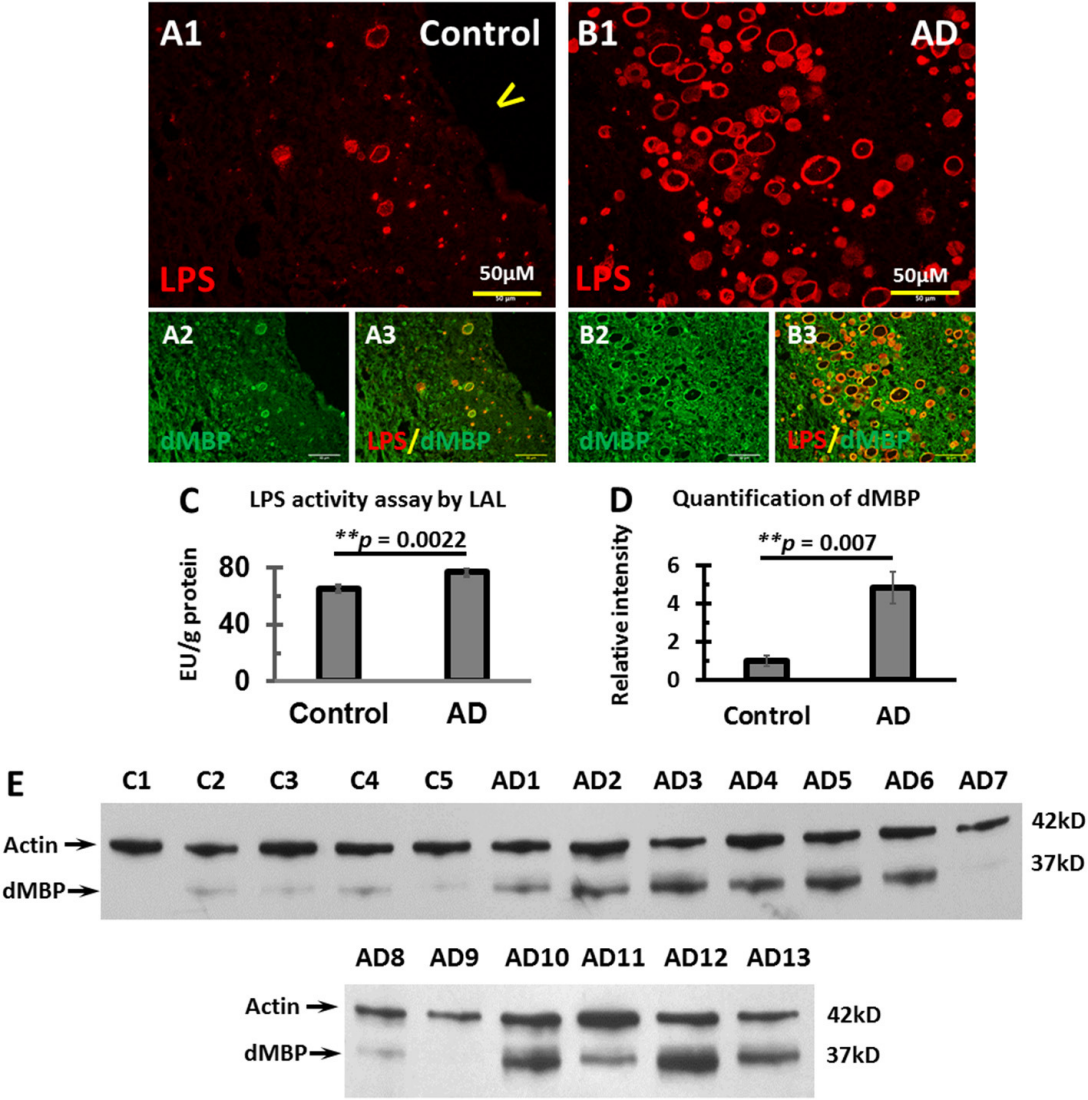
Colocalization of LPS with dMBP, LPS LAL assay, and Western blot analysis of dMBP in the PVWM of AD and control brains. **(A)** LPS **(A1)** and dMBP **(A2)** in control PVWM were colocalized **(A3)**. **(B)** LPS **(B1)** and dMBP **(B2)** in AD PVWM were colocalized **(B3)**. **(C)** The LAL assay for LPS of PVWM showed significantly greater LPS activity in AD (77.3 ± 3.1 EU/g, *n* = 12) compared to controls (65 ± 2.1 EU/g, *n* = 10, *p* = 0.0022). **(D)** The dMBP band intensity in PVWM was greater in AD (4.8 ± 0.8, *n* = 13) compared to controls (1 ± 0.3, *n* = 5, *p* = 0.007). **(E)** Western blots of PVWM for dMBP and β-actin for 5 controls (C1-C5) and 13 AD samples (AD1-AD13). β-actin was used as a loading control. Note: LAL, Limulus Amoebocyte Lysate assay for LPS; dMBP, degraded myelin basic protein; LPS, lipopolysaccharide; AD, Alzheimer's disease; Bar = 50 μm.

We, therefore, stained for LPS and dMBP in control brains (Figures 5A1–A3) and AD brains (Figures 5B1–B3). LPS colocalized with dMBP in the vesicles of control brains (Figure 5A3) and AD brains (Figure 5B3).

To quantify LPS levels in the PVWM, we performed the LPS LAL enzymatic assay for LPS from PVWM of AD and control brains. The data showed greater LPS activity in AD PVWM (77.3 ± 3.1 EU/g) compared to controls (65.0 ± 2.1 EU/g, *p* = 0.0022, Figure 5C). To quantify dMBP levels in the PVWM, we performed Western blots from PVWM of AD and control brains. The blots showed significantly more dMBP in AD PVWM compared to control (Figure 5E). Quantitative analysis confirmed the density of the dMBP bands in AD PVWM (4.8 ± 0.8) was much higher than in controls (1.0 ± 0.3) (Figure 5D, *p* = 0.007).

These data show that LPS co-localizes with dMBP in PVWM vesicles, and that both LPS and dMBP are increased in AD PVWM compared to controls. Whether LPS causes myelin degradation in the vesicles requires further study.

### Inflammation Related to LPS^+^ Vesicles

**Figure 6:**
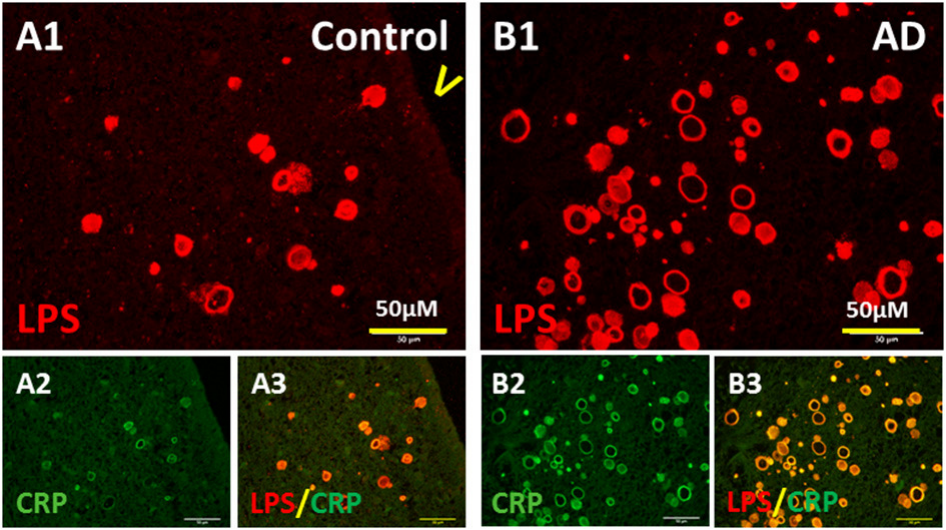
Colocalization of LPS with CRP. **(A)** LPS **(A1)** and CRP **(A2)** in control PVWM were co-localized **(A3)**. **(B)** LPS **(B1)** and CRP **(B2)** in AD PVWM were co-localized **(B3)**. Note: LPS, lipopolysaccharide; CRP, C-reactive protein; V, ventricle; PVWM, periventricular white matter; AD, Alzheimer's disease; Bar = 50 μm.

LPS are potent pro-inflammatory agents that provoke a strong immune response and induce many molecules including C-reactive protein (CRP). Thus, we stained PVWM for CRP and LPS. Indeed, CRP was found in the walls of the vesicles in MDZ of PVWM in both the control brain (Figure 6A2) and AD brain (Figure 6B2). CRP co-localized with LPS in vesicles in the MDZ in control (Figures 6A3,A1) and AD brains (Figures 6B3,B1). These data are consistent with the suggestion that LPS promotes inflammation manifested in part by the induction of CRP.

**Figure 7:**
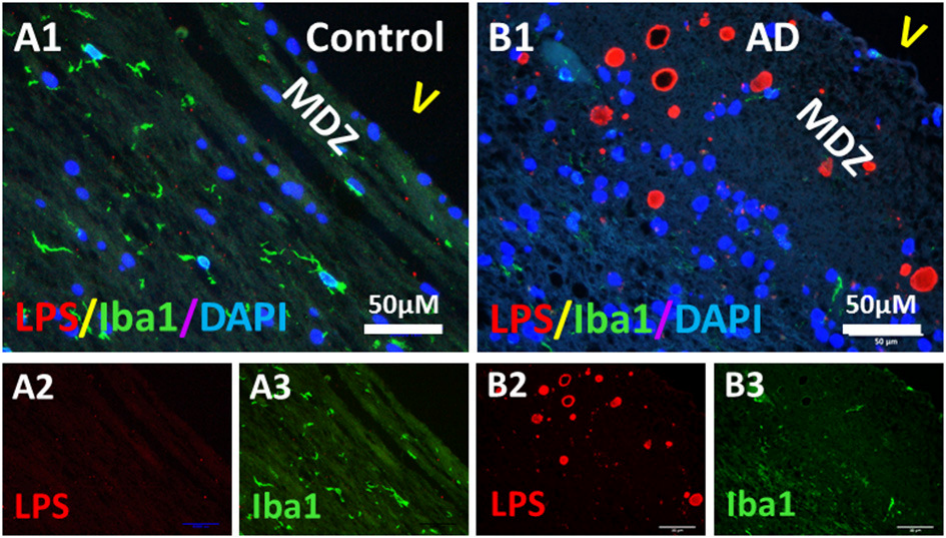
Double immunostaining of LPS and microglial Iba1 in the PVWM of AD and aging control brains. **(A)** LPS **(A2)** and Iba1 **(A3)** in control PVWM did not co-localize **(A1)**. Iba1 stained microglia were sparse **(A1,A3)**. **(B)** LPS **(B2)** and Iba1 **(B3)** in AD PVWM did not co-localize **(B1)**. Iba1 stained microglia were sparse in AD PVWM **(B1,B3)**. Note: MDZ, myelin deficient zone; V, ventricle; LPS, lipopolysaccharide; Iba1, microglial marker; AD, Alzheimer's Disease; Bar = 50 μm.

Since microglia and astrocytes are the resident immune cells that defend the host against infection or injury, and since bacterial LPS was detected in the PVWM of AD brains, we determined whether microglia and astrocytes were associated with LPS in the PVWM of AD brains compared to controls using the Iba1 microglial marker and the GFAP astrocyte marker. The results showed that Iba1 stained microglia were sparse in the myelin-deficit zone (MDZ) of the aging control brain that contained little LPS (Figures 7A1–A3). Unexpectedly, Iba1 stained microglia were also sparse in the MDZ zone of AD brains even around LPS^+^ vesicles (Figures 7B1–B3). Iba1 did not colocalize with LPS in either control (Figure 7A1) or AD (Figure 7B1) MDZ.

**Figure 8:**
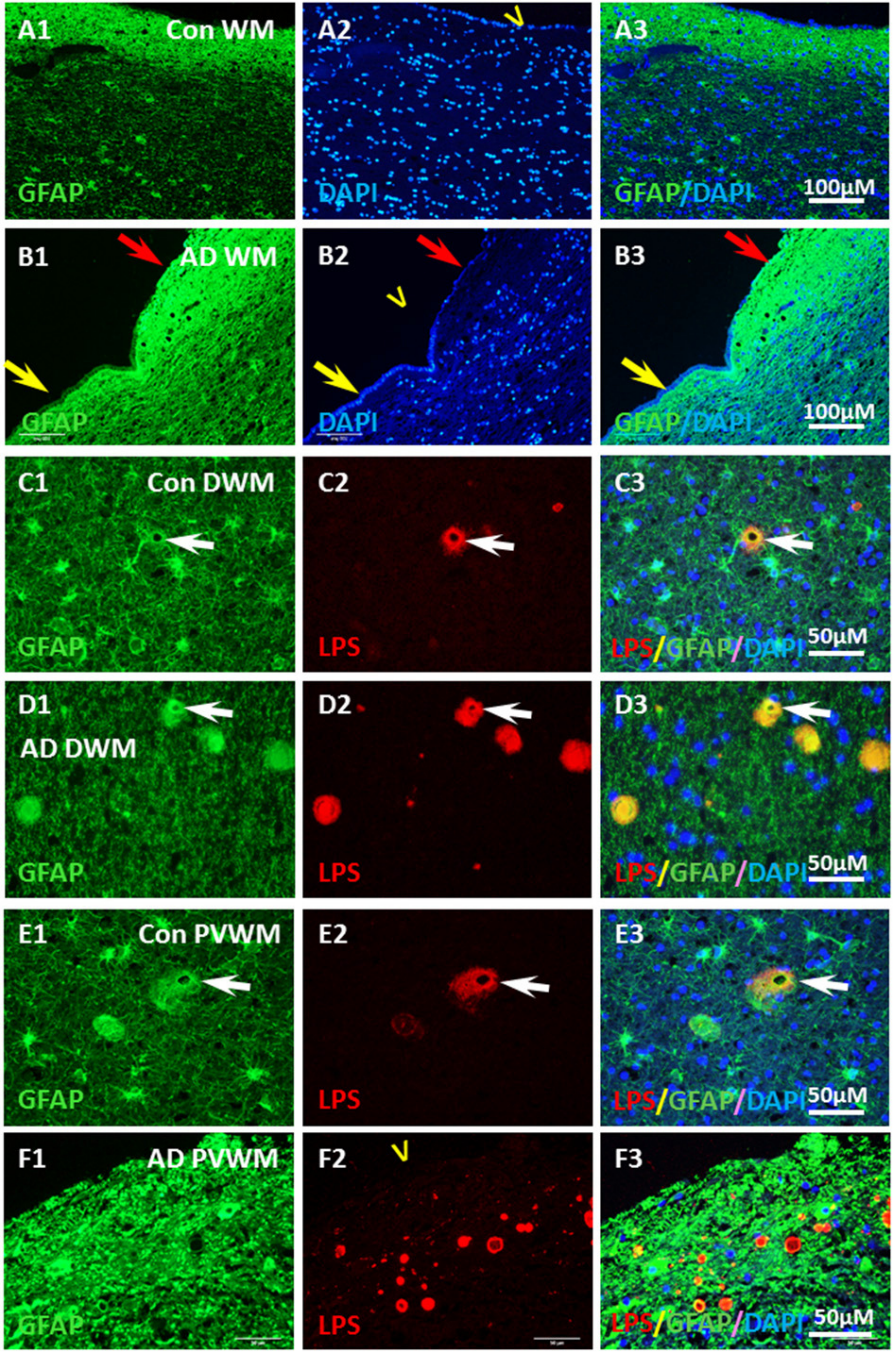
Localization of GFAP and LPS in the vesicles in AD and aging WM. **(A)** GFAP was intensely stained in the control PVWM myelin deficient zone, which forms a GFAP reactive layer immediately beneath the ependymal lining **(A1,A3)**. DAPI stained all nuclei in the section, including the ependymal cells **(A2,A3)**. **(B)** GFAP was intensely stained in the AD PVWM myelin deficient zone, which forms a GFAP reactive layer immediately beneath the ependymal cells **(B1,B3)**. The GFAP stained layer was thicker where ependymal cells were partially lost (**B1–B3**, red arrow) compared to areas where ependymal cells were intact (**B1–B3**; yellow arrow). DAPI staining showed loss of ependymal cells in some areas (**B2,B3** red arrow). **(C)** In control deep white matter (Con DWM), scattered astrocytes were observed **(C1)** a few of which stained for LPS **(C2,C3)**. **(D)** In Alzheimer's Disease deep white matter (AD DWM) scattered astrocytes **(D1)** were also observed, many of which stained for LPS (**D1–D3**, arrow). **(E)** In control periventricular white matter (Con PVWM) scattered astrocytes were observed **(E1)** some of which were stained for LPS (**E2,E3**, arrow). **(F)** In Alzheimer's Disease periventricular white matter (AD PVWM) there was dense GFAP staining throughout the myelin deficient zone **(F1)**. LPS staining showed discrete vesicles in this zone **(F2)**, the margins of which appeared to co-localize with or were immediately adjacent to GFAP positive astrocytic processes **(F3)**. Note: LPS, lipopolysaccharide; V, ventricle; WM, white matter; PVWM, periventricular white matter; DAPI, nuclear stain; DWM, deep white matter; AD, Alzheimer's disease; Bar = 100 μm in **(A,B)**; Bar = 50 μm in **(C–F)**.

In contrast, there was intense GFAP staining in the myelin-deficit zone of control brains (Figures 8A1–A3) and AD brains (Figures 8B1–B3). There were scattered astrocytes in the white matter adjacent to the MDZ in both control and AD brains. Some of the astrocytes in deep white matter in control brains (Figures 8C1,C3) and AD brains (Figures 8D1,D3) co-localized with LPS (Figures 8C2,C3,D2,D3). In the periventricular white matter, reactive astrocytes were observed both in control (Figures 8E1,E3) and AD (Figures 8F1,F3) brains and LPS was colocalized with GFAP or was immediately adjacent in control (Figures 8E2,E3) and AD (Figures 8F2,F3) brain. Negative control for GFAP immunostaining (deleting antibody to GFAP) showed no signal in PVWM of AD and control brains (Supplementary Figure 3). The data suggest an intense astrocyte response to the loss of myelin in the MDZ of both control and AD brains, but little ongoing microglial response.

### LPS^+^ Vesicles Stain for PAS and Are CA

**Figure 9:**
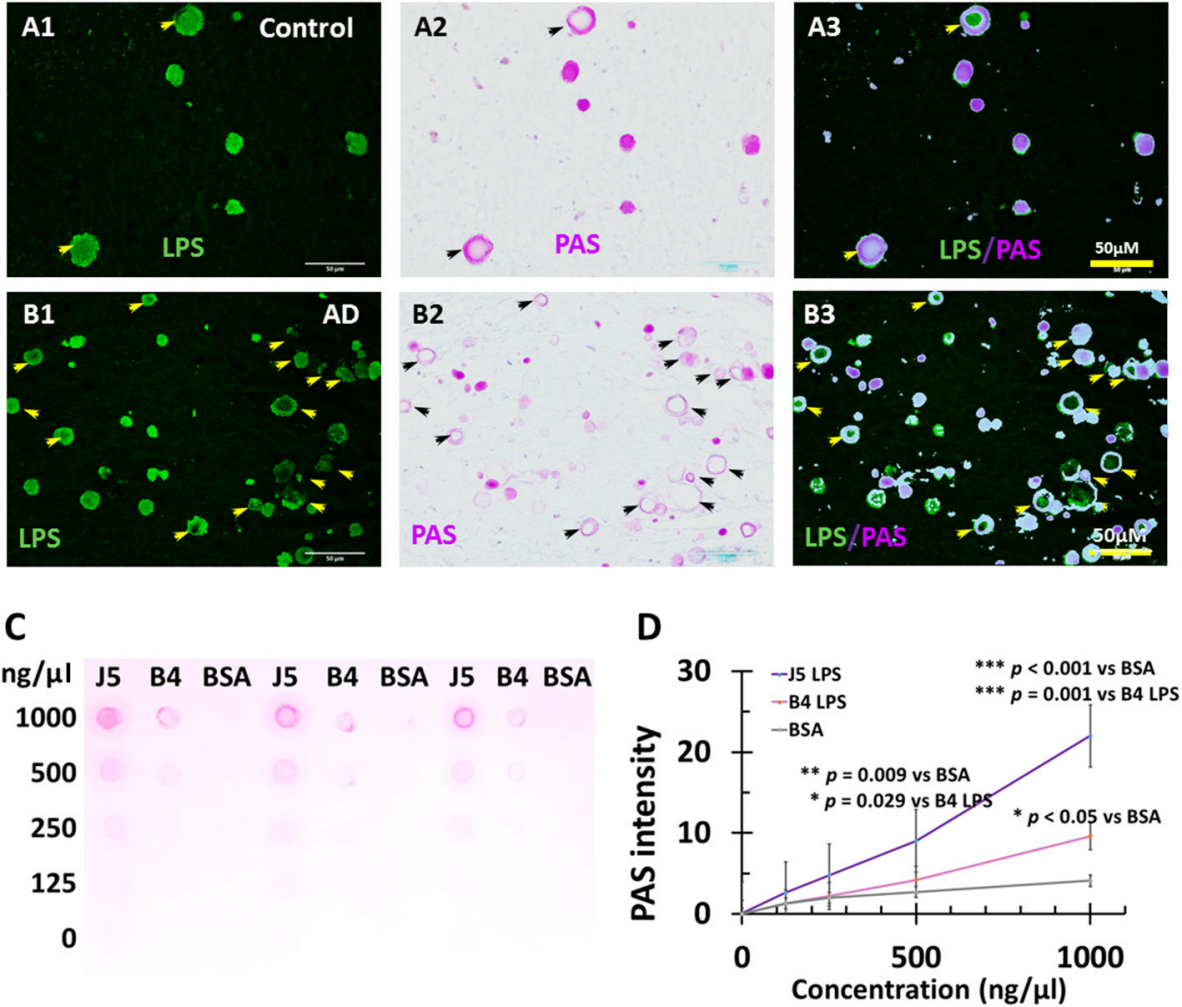
PAS staining of LPS in *Corpora amylacea* of AD and aging brains, and PAS staining of purified LPS. In control, PVWM LPS **(A1)** and PAS **(A2)** were co-localized **(A3)**. IN AD PVWM there were more LPS stained vacuoles **(B1)** and PAS-stained *Corpora amylacea* **(B2)** which were mostly co-localized **(B3)**. Most but not all LPS stained vesicles were PAS positive and therefore were *Corpora amylacea* (CA). PAS stained both *E. coli* J5 LPS and *E. coli* O111:B4 LPS at the higher concentrations (1,000 ng/μl and 500 ng/μl) with much greater staining intensity for *E. coli* J5 LPS than *E. coli* O111:B4 LPS at the same concentrations **(C,D)**. When the LPS concentrations were 250 ng/μl, PAS only stained *E. coli* J5 LPS but not the *E. coli* O111:B4 LPS. However, the difference was not significant. When the LPS concentrations were 125 ng/μl, PAS did not stain either *E. coli* J5 LPS or *E. coli* O111:B4 LPS. Note: LPS, lipopolysaccharide; PAS, Periodic acid–Schiff, a marker for CA; J5, *E. coli* J5 LPS; B4, *E. coli* O111:B4 LPS; AD, Alzheimer's disease; Bar = 50 μm.

The LPS^+^ or dMBP stained vesicles were morphologically similar to *corpora amylacea* which are usually identified by Periodic acid-Schiff (PAS) staining. Since PAS stains polysaccharides and LPS are important polysaccharides in the outer membrane of Gram-negative bacteria, we hypothesized that PAS might stain LPS. We, therefore, performed double staining for PAS and LPS in the myelin-deficient zone in the PVWM. The results showed LPS-stained vesicles in control (Figure 9A1) and AD brains (Figure 9B1), and PAS-stained CA in control (Figure 9A2) and AD brains (Figure 9B2). Overlay of the images showed that LPS was co-localized with PAS in the control (Figure 9A3) and AD brains (Figure 9B3), though some LPS vesicles did not contain PAS and some PAS-stained *corpora amylacea* were negative for LPS in AD brains (Figure 9B3).

These findings indicate that PAS probably does stain polysaccharides in bacterial LPS, and LPS staining vesicles constitute most of the *corpora amylacea* found in aging control and AD brains.

### Purified LPS Stains for PAS

To further confirm that PAS stains LPS, we performed PAS staining for purified LPS from *E. coli*, serotype O111:B4 and its mutant form of *E. coli*, serotype J5. The results showed that PAS did stain both *E. coli* J5 LPS and *E. coli* O111:B4 LPS at higher concentrations (1,000 ng/μl and 500 ng/μl) with much greater staining intensity for *E. coli* J5 LPS than *E. coli* O111:B4 LPS at the same concentrations (Figures 9C,D; *p* = 0.001 at 1,000 ng/μl and *p* = 0.029 at 500 ng/μl, respectively). When the LPS concentrations were 250 ng/μl, PAS only stained *E. coli* J5 LPS but not the *E. coli* O111:B4 LPS. However, the difference was not significant. When the LPS concentrations were 125 ng/μl, PAS did not stain either *E. coli* J5 LPS or *E. coli* O111:B4 LPS.

These findings suggest that PAS stains LPS in a dose-dependent and strain-dependent manner.

## Discussion

A myelin deficient zone was observed in PVWM in AD and control aging brains. This zone contained vesicles, many of which were corpora amylacea stained with PAS and co-localized with LPS. The CA/vesicles had evidence of oxidative stress to GALC and NG2, markers for oligodendrocytes (OLs), and oligodendrocyte precursor cells (OPCs), respectively. There were more LPS^+^ vesicles in AD than control PVWM, and LPS activity was greater in AD than control PVWM. The LPS^+^ vesicles were associated with the acute phase protein CRP, with degraded and oxidized myelin products, and were surrounded by astrocytes. Degraded MBP was found in the LPS^+^ vesicles and dMBP levels were significantly greater in AD compared to control PVWM. PAS, the CA marker, was shown to directly stain LPS in nitrocellulose membranes. We postulate that CA/LPS^+^ vesicles participate in a cerebral innate immune defense against LPS, damaged myelin, and other reactive molecules.

### Inflammation of PVWM in AD and Aging Brains

We have previously demonstrated that AD brains have significant loss of intact MBP and an increase in dMBP in PVWM adjacent to a denuded ependymal layer (Zhan et al., 2014). In regions of myelin loss, vesicles that stained positive for dMBP, myelin lipid, and neurofilament but not for intact MBP were identified (Zhan et al., 2014). AD brains have significantly more vesicles in the PVWM compared to control brains (Zhan et al., 2014). In addition, we have also demonstrated that LPS in AD gray matter and AD white matter were greater than in control brains (Zhan et al., 2016, 2018). In this study, we show that the dMBP containing vesicles also contained Gram-negative bacterial LPS and that LPS co-localized with PAS in most of the vesicles, identifying the LPS-dMBP stained vesicles as *corpora amylacea* (CA).

Lipopolysaccharides are the major components of the outer membrane of Gram-negative bacteria and are potent inflammatory agents. LPS has been identified in human blood (Klimiec et al., 2016, 2018; Hakoupian et al., 2021) and brains (Zhan et al., 2016, 2018) with higher levels of LPS in blood (Zhang et al., 2009) and brains (Zhan et al., 2016, 2018; Zhao et al., 2017) of AD compared to controls. CRP is a plasma acute-phase protein whose concentrations increase in response to inflammation. Our recent study has demonstrated that plasma LPS levels positively correlated with plasma CRP levels in humans (Hakoupian et al., 2021). Other studies demonstrated that CRP levels are elevated in blood years before the onset of AD (Schmidt et al., 2002; Engelhart et al., 2004), and plasma CRP levels are higher in patients with AD compared to controls (Song et al., 2015). Though CRP is not normally found in normal brains, it has been reported in the tangles (Duong et al., 1997), plaques (Iwamoto et al., 1994), and pyramidal neurons of AD brains (Yasojima et al., 2000). It is unclear whether CRP plays a role in AD pathogenesis, or is simply a response to LPS and other pro-inflammatory molecules in the AD brain.

How LPS entered brain PVWM is still unclear. However, we have previously found that the ependymal cells were injured in aging brains and that the ependymal cell loss was more severe in AD compared to controls brains (Zhan et al., 2014). Most of the LPS in deep regions of the brain was co-localized with the vesicles in the myelin deficient zone adjacent to the ependymal layer. This finding suggests that ependymal injury might be associated with LPS entry into the PVWM which could be carried into the PVWM by CSF from the lateral ventricles.

The myelin injury was associated with oxidative damage. Degraded MBP was co-localized with LPS in the vesicles located in the myelin-deficit zone of aging and AD brains. Higher levels of LPS and dMBP were found in AD than controls. LPS was also associated with oxidized myelin molecules in the vesicles. We speculate that the LPS contributed to the oxidative stress that caused the ependymal and myelin injury.

Glial fibrillary acidic protein positive astrocytes were intensely stained in the myelin-deficit zone that was adjacent to the lateral ventricles. Astrocytes are involved in forming the blood-brain barrier (BBB) which modulates the passage of molecules including nutrients, ions, glucose, water, and amino acids and restricts the passage of pathogens. Since the astrocytes appeared to be intimately associated with the walls of the vesicles, we postulate they might form a barrier to help prevent bacterial components such as LPS from spreading to other parts of the brain.

We have previously shown that Iba1^+^ microglia associate with LPS in the gray matter of AD brains (Zhan et al., 2016). Activated microglia are involved in AD pathology in gray matter and amyloid plaques (Dickson et al., 1988; Serrano-Pozo et al., 2013; Zhan et al., 2016; Hansen et al., 2018; Ahmad et al., 2019; Dionisio-Santos et al., 2019). We expected increased numbers of activated microglia in areas of myelin injury in the PVWM of AD brains. Surprisingly, Iba1^+^ microglia were quite sparse in the PVWM of aging and AD brains. Presumed microglial dysfunction has been suggested in frontotemporal lobar degeneration (Sakae et al., 2019) and AD (Piirainen et al., 2017; Yoshino et al., 2017; Andreone et al., 2020; Gabande-Rodriguez et al., 2020). Alternatively, it is possible that the phagocytic function of the microglia has already been completed with the removal of most of the injured myelin, compartmentation of damaged myelin, and LPS in the vesicles resulting in little ongoing microglial activity at this point.

Lipopolysaccharides were localized to microglia, neurons, OPCs, oligodendrocytes, and extracellular amyloid plaques in AD gray matter in our previous study (Zhan et al., 2016). We also found Gram-negative bacterial DNA in AD and aging brains. Recent studies demonstrated that bacterial DNA promotes aggregation of β-amyloid and tau, two hallmarks of AD pathology (Tetz et al., 2020; Tetz and Tetz, 2021). The DNA was derived from *Porphyromonas gingivalis, Burkholderia burgdorferi*, and different strains of *E. coli*, all of which are gram-negative and have been associated with AD (Tetz et al., 2020). Other bacteria and viruses have also been linked to AD pathology (Hashioka et al., 2008; Hammond et al., 2010; Miklossy, 2011, 2015, 2016; Lim et al., 2014; Fulop et al., 2018). These microorganisms and their molecular components are derived from the gut, skin, gums, and other organs, and presumably cross the BBB and might play a role in the pathogenesis of AD. This study supports the existence of some role for LPS in AD pathogenesis since it is localized to dMBP-PAS positive vesicles which appear to be CA.

### LPS Positive Vesicles Are CA and PAS Directly Stains LPS

The vesicles described in the myelin deficient zone adjacent to the ventricles appear mostly to be CA in both control and AD brains. Therefore, the vesicles and CA might have similar functions. Based upon the molecules found in the vesicles/CA, possible functions include being part of the innate immune response, inflammation, oxidative stress, myelin degradation, and compartmentalizing microbial molecules. The findings suggest that CA are waste depots where deleterious molecules like LPS are deposited, and damaged organelles and damaged myelin are restricted by actions of the innate immune system (Auge et al., 2017; Riba et al., 2019).

Lipopolysaccharides were co-localized with PAS in many vesicles of AD and control brains. Since PAS is a staining method used to detect polysaccharides in CA, and LPS are important bacterial cell-surface polysaccharide, it is possible that LPS stained positive for PAS in CA in aging control and AD brains. In AD brains, most LPS^+^ vesicles stained positive for PAS, which suggests that the LPS in the vesicles can be detected by PAS staining. However, some PAS-stained CA did not contain LPS, which suggests that other polysaccharides other than LPS might be present. In addition, some LPS positively stained vesicles did not stain for PAS, which suggests that not all LPS can be detected by PAS.

In our previous studies, LPS was detected by immunostaining, which involves antibody signal detection and amplification that can detect very small amounts of LPS. We hypothesized that even if PAS does stain LPS, perhaps it does not stain low levels of LPS. This hypothesis was confirmed by the PAS staining of purified LPS on nitrocellulose membranes at different concentrations. PAS stained both *E. coli* J5 LPS and *E. coli* O111:B4 LPS at high concentrations of LPS (1000 ng/μl and 500 ng/μl) with much greater staining intensity for *E. coli* J5 LPS than *E. coli* O111:B4 LPS at the same LPS concentration. When LPS concentrations were 125 ng/μl, PAS did not stain either *E. coli* J5 LPS or *E. coli* O111:B4 LPS. These findings show that PAS stains LPS in a dose-dependent manner.

One notable finding was that PAS-stained *E. coli* J5 LPS more intensely than *E. coli* O111:B4 LPS at the same concentrations of LPS. *E. coli* J5 LPS is a mutant form of *E. coli* O111:B4 LPS lacking the O-antigen. Therefore, the molecular mass of *E. coli* J5 LPS is smaller than *E. coli* O111:B4 LPS. Thus, more LPS molecules are in *E. coli* J5 LPS solution than in *E. coli* O111:B4 LPS solution given a certain concentration and volume. This might contribute to the higher PAS staining intensity of *E. coli* J5 LPS than *E. coli* O111:B4 LPS. Even lacking the O-antigen (O-polysaccharide), *E. coli* J5 LPS was still stained by PAS, which suggests that O-antigen is not the key element that is recognized by PAS. These findings revealed a previously unknown feature of CA that PAS does stain LPS on nitrocellulose membranes, and therefore bacterial LPS is likely at least one of the polysaccharides stained by PAS in CA.

In summary, myelin injury occurs in the PVWM of aging brains and more so, in AD brains. Degraded MBP levels are greater in AD brains compared to controls. The myelin injury in aging control and AD brain was associated with oxidative damage and neuroinflammation that may be due, in part, to bacterial LPS. LPS activity was greater in AD compared to control PVWM, and LPS^+^ vesicles were more abundant in AD compared to control PVWM. Bacterial LPS was one of the polysaccharide sources in CA and PAS stained LPS on nitrocellulose membranes in a dose-dependent and strain-dependent manner. CA found in AD and aging control brains are associated with bacterial LPS and the innate immune response.

## Limitations

This is a preliminary postmortem study on a limited number of sections from a limited number of brains. This is primarily an observational study that presents some novel findings. Though we attempted to infer mechanisms that could have produced the pathology, there is no way to determine cause and effect based upon the findings reported here. Future mechanistic studies will be required to better understand the significance of these results to normal aging and AD.

A vesicle is often a subcellular structure, bounded by a membrane. We chose to use the term “vesicle” simply because it was convenient. It is possible that all of the vesicles described here are all CA, in which case *corpora amylacea* would be the preferred name because of the history and many studies of CA.

The monoclonal antibody to LPS used in this study does seem to bind LPS since immunoprecipitation of the antibody with LPS eliminated all immunostaining. As pointed out in our prior studies, however, this antibody produces a single band on Western blots that is greater than the expected molecular weight of LPS. Thus, we have suggested that the antibody likely detects LPS bound to another unknown molecule. Thus, we used a completely independent LAL enzymatic LPS assay to show LPS activity was higher in AD PVWM compared to controls.

Future studies will be needed to explore the birth and death of oligodendrocytes and OPCs in the myelin deficient zone and in areas adjacent to the MDZ. It is likely the MDZ region studied here is identical to what is referred to as periventricular White Matter Hyperintensities (WMH) on human MRI brain scans. This needs to be confirmed by comparing the brain pathology and MRI in individual cases in the future.

## Data Availability Statement

The raw data supporting the conclusions of this article will be made available by the authors, without undue reservation.

## Ethics Statement

The studies involving human participants were reviewed and approved by University of California at Davis Institutional Review Board. The patients/participants provided their written informed consent to participate in this study.

## Author Contributions

XZ designed the studies and wrote the manuscript. MH performed experiments under the supervision of XZ. L-WJ provided samples. L-WJ and FS reviewed subjects for correct diagnosis. MH and XZ performed statistical analysis. All authors made changes to the manuscript.

## Funding

These studies were supported by grants from the California Department of Public Health (16-10324 to FS and XZ; 18-10924 to XZ), a CART grant (Coins for Alzheimer's Research Trust to FS), and the National Institutes of Health (AG069815 to XZ and FS).

## Conflict of Interest

The authors declare that the research was conducted in the absence of any commercial or financial relationships that could be construed as a potential conflict of interest.

## Publisher's Note

All claims expressed in this article are solely those of the authors and do not necessarily represent those of their affiliated organizations, or those of the publisher, the editors and the reviewers. Any product that may be evaluated in this article, or claim that may be made by its manufacturer, is not guaranteed or endorsed by the publisher.

## Supplementary Material

Supplementary Figure 1 Autofluorescence removal test. At red fluorochrome wavelength, autofluorescence was detected in the PVWM of aging brains including control **(A1)** and AD **(B1)** brains. Similarly, at green fluorochrome wavelength, autofluorescence was detected in the PVWM of control **(C1)** and AD **(D1)** brains. After applying Autofluorescence Removal Reagent, fluorescent signals at red fluorochrome wavelength in control **(A2)** and AD **(B2)** were nearly eliminated. Similarly, after applying Autofluorescence Removal Reagent, fluorescent signals at green fluorochrome wavelength in control **(C2)** and AD **(D2)** were nearly eliminated. These data indicated that immunofluorescent signals detected in this study were not due to autofluorescence. Note: Auto^+^, Autofluorescence before removal; Auto^−^, Autofluorescence after removal; AD, Alzheimer's disease; PVWM, periventricular white matter; Bar = 100 μm.

Supplementary Figure 2 Negative control test for immunofluorescence. A negative control test for immunofluorescence was carried out the same as the regular immunofluorescence used in the study except primary antibodies were omitted. There were no fluorescence signals either at the red fluorochrome wavelength in control **(A1)** or in AD **(B1)** PVWM or at the green fluorochrome wavelength in control **(A2)** or AD **(B2)** PVWM. These data indicate the immunofluorescence signals were not due to non-specific staining from the secondary antibodies. Note: AD, Alzheimer's disease; PVWM, periventricular white matter; Bar = 100 μm.

Supplementary Figure 3 Immunofluorescence of GFAP and its negative control. Immunofluorescence of GFAP was detected in control PVWM **(A1)** and AD PVWM **(B1)**. In controls, GFAP positively stained astrocytes as well as the vesicle (**A1**, arrow). In AD PVWM **(B1)**, GFAP positively stained astrocytes and vesicles (**B1**, arrows) as well. Negative controls with deletion of primary GFAP antibody showed no signals in both control **(A2)** and AD **(B2)** brains. Note: AD, Alzheimer's disease; PVWM, periventricular white matter; Bar = 50 μm.
