# Supplementary figures and images for: Lipopolysaccharide, Identified Using an Antibody and by PAS Staining, Is Associated With Corpora amylacea and White Matter Injury in Alzheimer's Disease and Aging Brain
Source: Front Aging Neurosci. 2021 Nov 24;13:705594. doi: 10.3389/fnagi.2021.705594 (PMC8652352; doi:10.3389/fnagi.2021.705594)

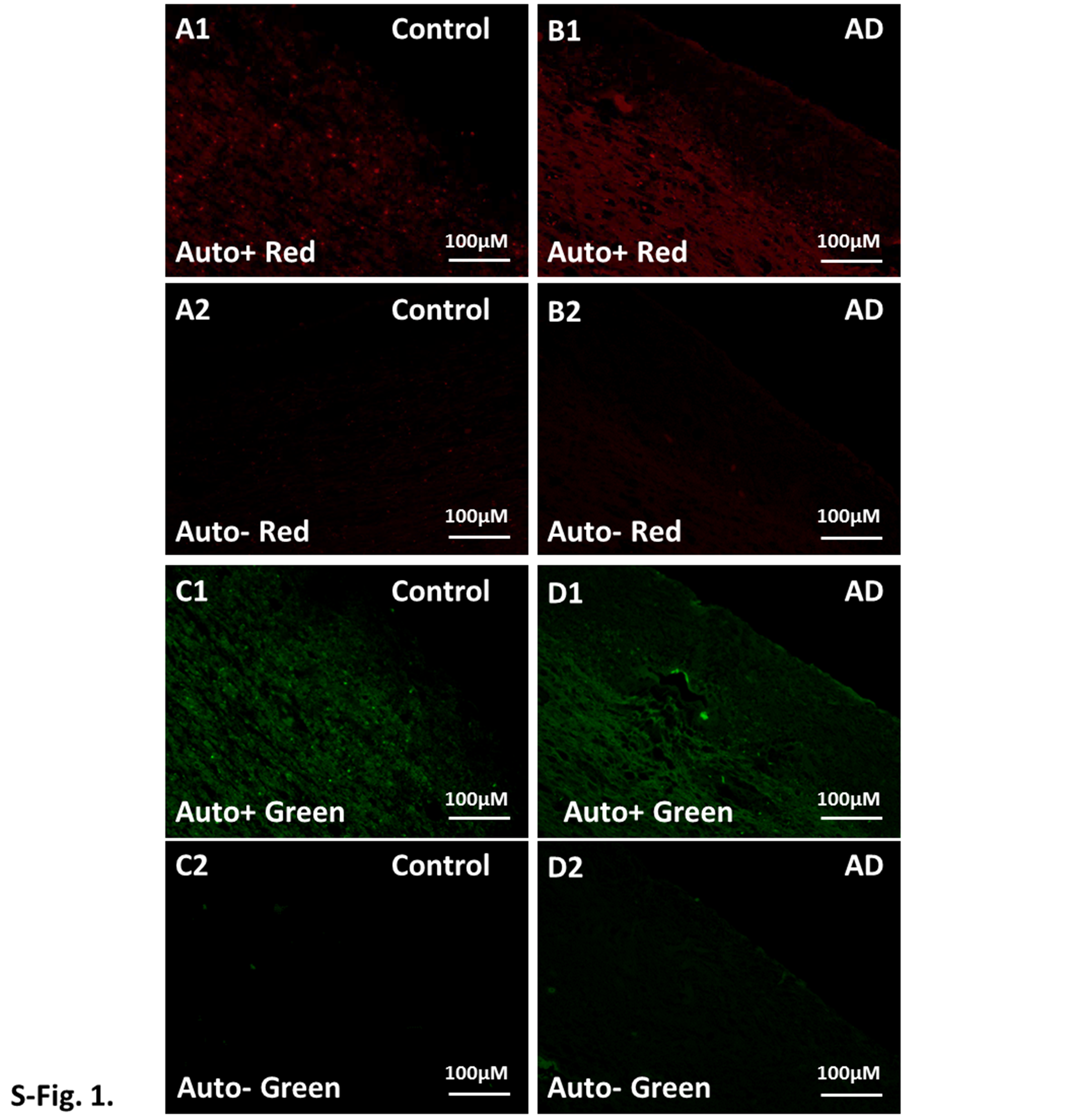

Supplement: Supplementary Figure 1 — Autofluorescence removal test. At red fluorochrome wavelength, autofluorescence was detected in the PVWM of aging brains including control (A1) and AD (B1) brains. Similarly, at green fluorochrome wavelength, autofluorescence was detected in the PVWM of control (C1) and AD (D1) brains. After applying Autofluorescence Removal Reagent, fluorescent signals at red fluorochrome wavelength in control (A2) and AD (B2) were nearly eliminated. Similarly, after applying Autofluorescence Removal Reagent, fluorescent signals at green fluorochrome wavelength in control (C2) and AD (D2) were nearly eliminated. These data indicated that immunofluorescent signals detected in this study were not due to autofluorescence. Note: Auto+, Autofluorescence before removal; Auto−, Autofluorescence after removal; AD, Alzheimer's disease; PVWM, periventricular white matter; Bar = 100 μm. [file Image_1.tif]

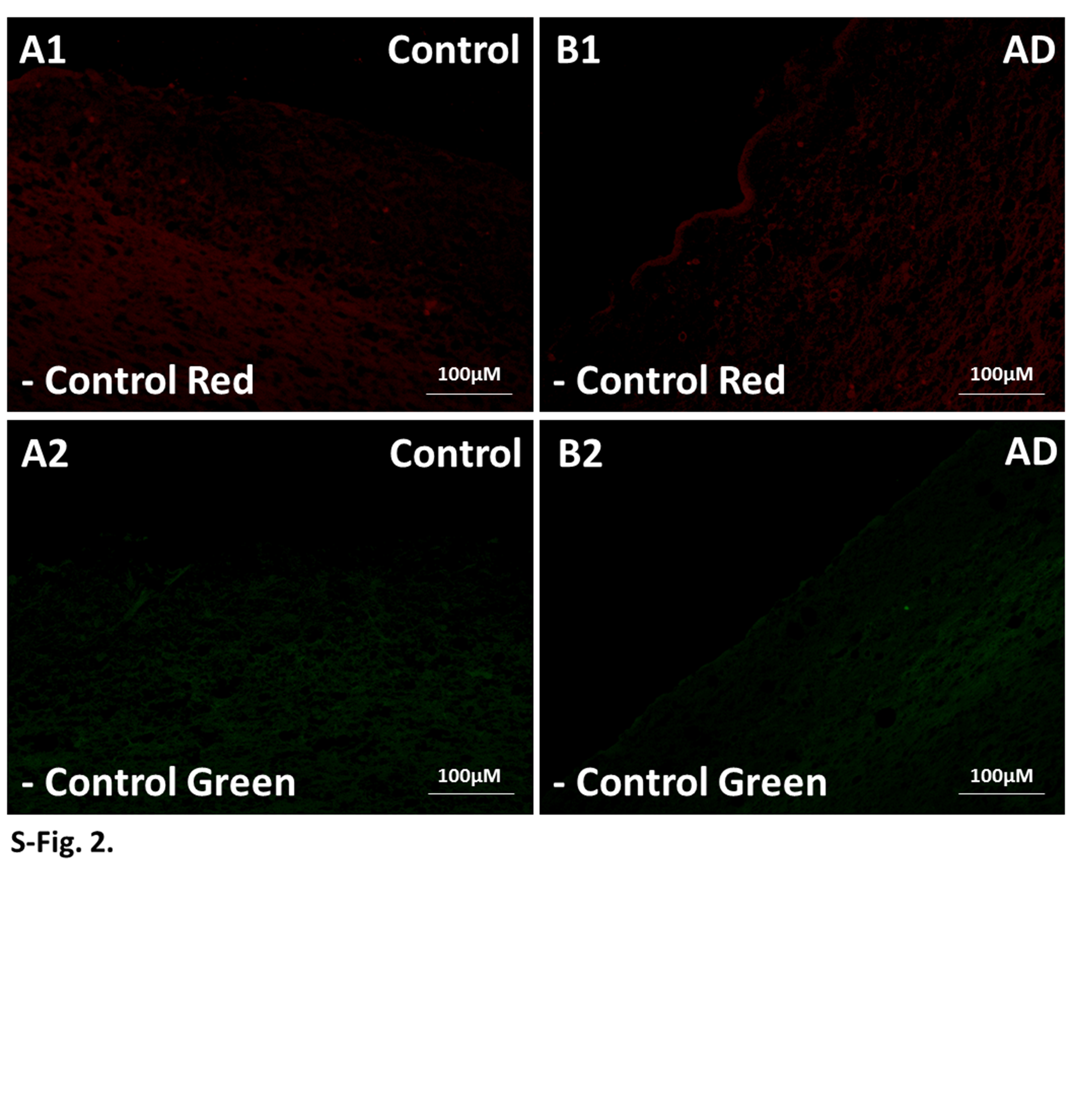

Supplement: Supplementary Figure 2 — Negative control test for immunofluorescence. A negative control test for immunofluorescence was carried out the same as the regular immunofluorescence used in the study except primary antibodies were omitted. There were no fluorescence signals either at the red fluorochrome wavelength in control (A1) or in AD (B1) PVWM or at the green fluorochrome wavelength in control (A2) or AD (B2) PVWM. These data indicate the immunofluorescence signals were not due to non-specific staining from the secondary antibodies. Note: AD, Alzheimer's disease; PVWM, periventricular white matter; Bar = 100 μm. [file Image_2.tif]

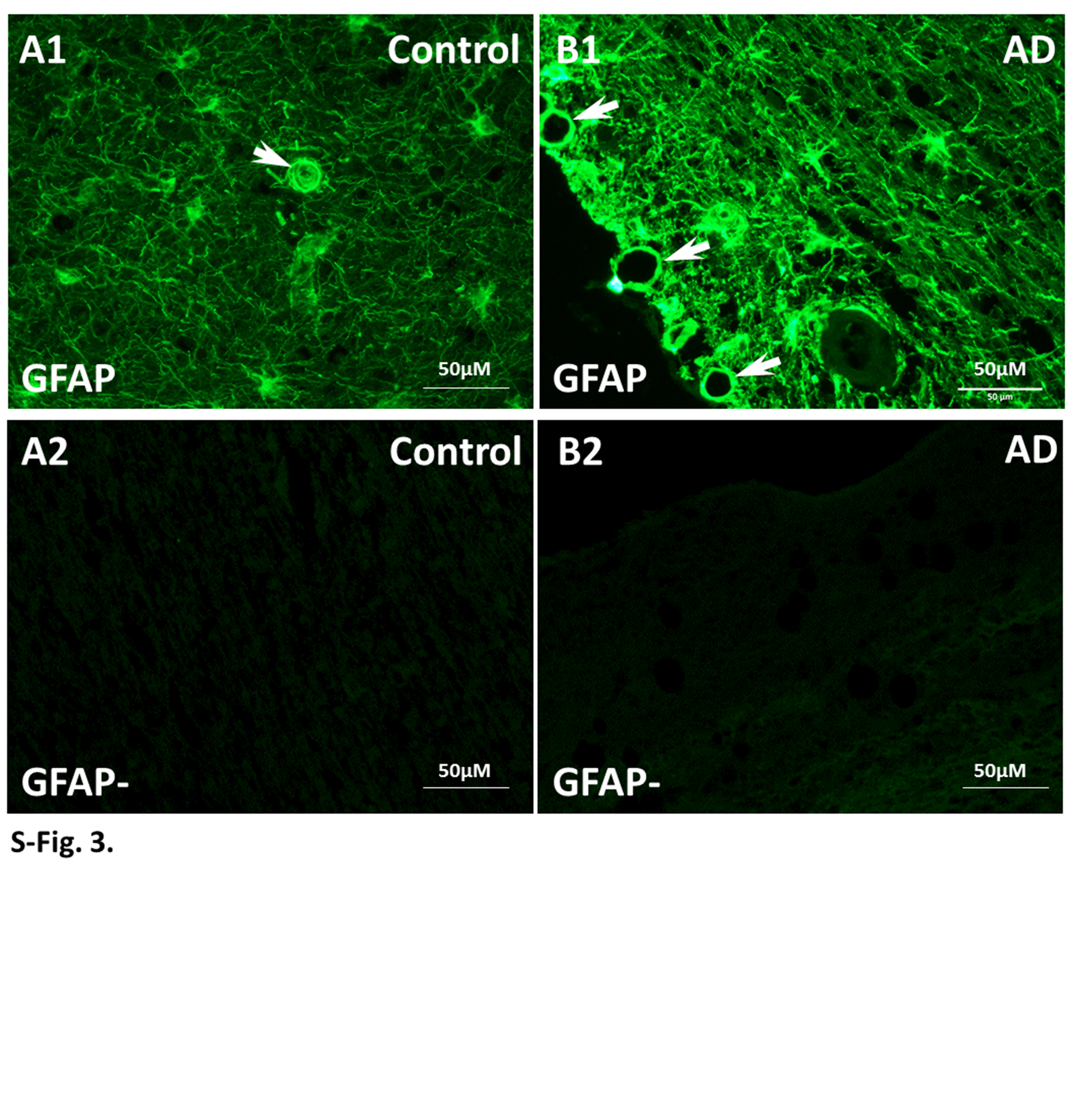

Supplement: Supplementary Figure 3 — Immunofluorescence of GFAP and its negative control. Immunofluorescence of GFAP was detected in control PVWM (A1) and AD PVWM (B1). In controls, GFAP positively stained astrocytes as well as the vesicle (A1, arrow). In AD PVWM (B1), GFAP positively stained astrocytes and vesicles (B1, arrows) as well. Negative controls with deletion of primary GFAP antibody showed no signals in both control (A2) and AD (B2) brains. Note: AD, Alzheimer's disease; PVWM, periventricular white matter; Bar = 50 μm. [file Image_3.tif]
